# Multi-scale environmental drivers of pollinator community composition, richness, and abundance across multiple taxa

**DOI:** 10.7717/peerj.21629

**Published:** 2026-09-30

**Authors:** Tea Skendzic, Snezana Popov, Marija Milicic, Marina Jankovic Milosavljevic, Snezana Radenkovic, Ante Vujic, Dusanka Vujanovic

**Affiliations:** 1 Department of Biology and Ecology, Faculty of Sciences, University of Novi Sad, Novi Sad, Vojvodina, Serbia; 2 BioSense Institute—Research and Development Institute for IT in Biosystems, University of Novi Sad, Novi Sad, Vojvodina, Serbia

**Keywords:** Pollinator communities, Wild bees, Hoverflies, Butterflies, Landscape composition, Transect characteristics

## Abstract

Pollination is a crucial ecosystem service sustaining plant reproduction, biodiversity, and ecosystem functioning. Understanding how pollinator communities respond to environmental factors is therefore essential for supporting pollinator services, particularly in agricultural landscapes. We surveyed three pollinator taxa, wild bees, hoverflies and butterflies, across 26 semi-arid grassland sites in Vojvodina (Serbia) during three seasons in 2022. We assessed how landscape-scale (agricultural, forest, and grassland cover) and local habitat variables (total flower cover and vegetation height) structure species composition, and how these variables, together with short-term weather conditions, influence species richness and abundance. Redundancy analysis (RDA) was used to assess drivers of community composition, while generalized additive models (GAMs) were applied to model richness and abundance patterns. Because observed richness was correlated with total abundance, we additionally conducted sensitivity analyses using Fisher’s alpha for wild bees and butterflies and Shannon diversity for hoverflies. Species composition in all three taxa was primarily structured by landscape-scale variables. Wild bee, hoverfly, and butterfly assemblages differed in their responses to landscape gradients, likely reflecting variation in life-history traits, dispersal, and larval ecology. In contrast, richness and abundance reflected additional fine-scale and short-term processes. Observed wild-bee richness was influenced by grassland cover and weather (temperature and wind speed), whereas Fisher’s alpha indicated a stronger role of vegetation height. Hoverfly richness was associated with local floral resources and weather, but these relationships were not retained in Shannon diversity models, suggesting that they partly reflected variation in abundance, activity, or detectability. Butterfly richness was associated with cloud cover, a pattern also supported by Fisher’s alpha. Weather variables affected observed richness despite surveys being conducted under standardized meteorological thresholds, demonstrating that biologically meaningful variation in pollinator activity and detectability can persist within accepted monitoring protocols. Pollinator responses were taxon-specific and differed between landscape and local environmental drivers, with landscape context shaping community composition and local habitat or weather conditions influencing richness, abundance, and activity-related patterns. Sensitivity analyses highlighted the importance of distinguishing observed richness from abundance-independent diversity, particularly for wild bees and hoverflies. Effective monitoring and conservation strategies should therefore integrate landscape context, local habitat quality, and weather covariates, while interpreting observed richness cautiously when abundance and detectability vary among sites.

## Introduction

Pollinators play a vital role in ecosystems, making them a crucial component of global biodiversity (Potts et al., 2016). Approximately 87.5% of flowering plant species are pollinated by animals, the majority of which are insects (Ollerton, Winfree & Tarrant, 2011). By facilitating pollen transfer, they sustain wild plant communities (Ashman et al., 2004; Aguilar et al., 2006) and enhance the yields and stability of many crops (Klein et al., 2007; Ricketts et al., 2008), underscoring their ecological and economic importance. Among insect pollinators, the orders Hymenoptera, Diptera, Coleoptera, and Lepidoptera play the most significant roles in plant pollination (Abrol, 2012).

Pollinator declines have drawn increasing concern since the late 20th century (Zattara & Aizen, 2021), with negative trends in wild pollinators now recognized globally (Potts et al., 2010; Goulson et al., 2015; Janousek et al., 2023). Multiple, often interacting drivers of pollinator population decline are implicated, including landscape conversion of natural habitats to urban and agricultural uses (Kennedy et al., 2013; Vanbergen & Insect Pollinators Initiative, 2013), pathogens (Colla et al., 2006; Purkiss & Lach, 2019), non-native species, and climate change (González-Varo et al., 2013; Martinet et al., 2021). Because these pressures act through habitat area, configuration, and resource dynamics, community responses must be evaluated at multiple levels (species composition, richness, abundance).

Composition (which species occur and how they reorder along gradients) helps anticipate ecosystem responses to disturbance and environmental change (Carrick & Forsythe, 2020). Richness reflects the breadth of the assemblage, whereas abundance indicates how strongly local resources and demographic processes sustain populations (Westphal, Steffan-Dewenter & Tscharntke, 2003; Verberk, 2011; Magurran, 2021). Considering all three dimensions provides a fuller picture of how environmental variables shape pollinator communities and strengthens conservation planning and trend assessment.

Despite extensive research on pollinator declines, identifying consistent drivers of community patterns across taxa and spatial scales remains a key challenge. Although multi-scale approaches are increasingly used (Holland & Yang, 2016; Betts, Hadley & Kormann, 2019; Gathof et al., 2022), many studies still focus on single taxonomic groups or a limited set of environmental drivers, which constrains understanding of how landscape context, local habitat structure, and short-term weather jointly influence pollinator communities. Comparative, multi-taxon assessments that integrate landscape, local, and atmospheric drivers within a unified analytical framework remain relatively scarce, particularly in intensively managed agricultural regions of southeastern Europe. Addressing these gaps is essential for developing generalizable ecological inference and effective habitat-management strategies.

The present study focuses on three pollinator taxa, wild bees (Hymenoptera: Anthophila), hoverflies (Diptera: Syrphidae), and butterflies (Lepidoptera: Rhopalocera), which differ markedly in mobility, life history, larval ecology, and resource requirements. By examining multiple taxa simultaneously, we are able to distinguish shared environmental drivers from taxon-specific responses, thereby strengthening ecological inference and improving habitat-management recommendations. Because environmental drivers of pollinator communities operate at complementary spatial scales, we analyzed predictors at both the landscape and transect levels. The landscape scale (1 km radius) captures overall habitat availability and broader environmental context, which are known to influence pollinator community composition and population persistence (Osborne et al., 2007; Meyer, Jauker & Steffan-Dewenter, 2009). In contrast, the transect scale reflects local floral resources and vegetation structure that pollinators can directly access during foraging, providing an estimate of immediate food availability and microhabitat conditions (Steffan-Dewenter et al., 2002; Tscharntke et al., 2005). Given that many solitary bees forage within a few hundred meters, while bumblebees, hoverflies, and butterflies can forage over distances approaching or exceeding 1 km, the 1-km landscape buffer captures the broader habitat mosaic relevant to more mobile taxa (Osborne et al., 2007; Meyer, Jauker & Steffan-Dewenter, 2009; Nowicki et al., 2013), while transect-level variables reflect the fine-scale resource conditions experienced during foraging.

We focus on three dominant land-cover variables: grassland, agricultural, and forest cover, which represent the principal gradients of habitat-availability and land-use intensity structuring pollinator assemblages in agricultural landscapes. Habitats such as grasslands and forests provide complementary nesting substrates, larval resources, and season-long floral continuity. Their extent and connectivity are consistently associated with higher pollinator diversity and stability (Öckinger & Smith, 2007; Ulyshen et al., 2023; Ranalli et al., 2025; Sloan et al., 2025). Although agricultural sites may temporarily supply nectar and pollen through mass-flowering crops and field margins (Ulyshen et al., 2023), agricultural intensification, characterized by high agrochemical inputs (Whitehorn et al., 2012; Peterson et al., 2021), frequent tillage (Santos et al., 2024), large field sizes (Tscharntke et al., 2005), and simplified crop rotations (Benton, Vickery & Wilson, 2003), often erode these potential benefits. Under intensive management, floral resources tend to be taxonomically impoverished and short-lived, while nesting and larval habitats decline. In addition, both direct and indirect mortality risks increase, resulting in an overall negative effect on pollinators (Potts et al., 2010; Holzschuh et al., 2011; Kennedy et al., 2013; Goulson et al., 2015; Dainese et al., 2019).

At the transect scale, we included variables capturing resource availability and habitat structure. Total flower cover represents immediate nectar and pollen availability (Koupilová, Štenc & Janovský, 2022), and is a standardized proxy for the local availability of floral resources (Kleijn & Van Langevelde, 2006; Alison et al., 2021; Biegerl et al., 2025), while vegetation height serves as a proxy for management intensity and structural complexity, affecting the accessibility of inflorescences and local microclimatic conditions (Klecka, Hadrava & Koloušková, 2018). Green vegetation cover reflects overall vegetative continuity and structural buffering, influencing microclimate regulation, shelter provision, and the presence of nesting or larval substrate (Süle et al., 2023). Together, these local variables capture fine-scale habitat conditions that may modify or mediate landscape-level effects.

In addition to spatial habitat structure, short-term weather conditions strongly influence pollinator activity and detectability (Vicens & Bosch, 2000; Mahon & Hodge, 2022; Coates et al., 2024), even when surveys are conducted under standardized meteorological protocols. As ectotherms, pollinators rely on ambient temperature to regulate metabolic activity and flight performance (Kenna, Pawar & Gill, 2021; Johnson et al., 2023; Devenish et al., 2024). Wind can reduce flight efficiency and foraging success, while cloud cover alters solar radiation and microclimatic conditions near vegetation. Consequently, weather affects both realized activity and observed abundance, making it essential to account for short-term atmospheric variation when disentangling habitat effects from activity-driven differences.

In this study, we (1) examine how variables at the landscape and transect scales influence pollinator species composition for each taxon separately, and (2) assess the independent effects of these variables, along with short-term weather conditions, on pollinator richness and abundance. Landscape structure reflects the baseline availability of suitable habitats, local variables capture fine-scale resource availability and structural conditions, and weather conditions may influence the number of individuals detected during surveys through activity-mediated processes.

Given the marked differences among wild bees, hoverflies, and butterflies in mobility, larval ecology, nesting requirements, and thermal sensitivity (Ganuza et al., 2022; Vujanović et al., 2023; Bottero et al., 2023), we expected taxon-specific responses to environmental variables. Specifically, we predicted that wild bees would respond more strongly to local habitat structure and floral-resource availability because many species have limited foraging ranges and nesting requirements closely linked to local vegetation conditions. In contrast, hoverflies and butterflies, which generally have greater dispersal capacity, were expected to respond more strongly to broader landscape composition. We also expected short-term weather conditions to influence observed richness and abundance in a taxon-specific manner, despite surveys being conducted under standardized weather thresholds, because pollinator taxa differ in thermal sensitivity and behavioral responses to temperature, wind, and cloud cover.

## Materials and Methods

### Study area and species data

Pollinators were surveyed across 26 sites (Fig. 1) representing semi-arid grasslands located in the northern part of Serbia (Vojvodina province), a predominantly agricultural area with fertile arable land covering approximately 84% of the province (Sredojevic, 2014), whereas forest and semi-natural habitats comprise only about 10.8% of the area (Marković et al., 2020). Sites were separated by at least 2 km to reduce potential spatial autocorrelation. Using Corine Land Cover data (2018) in combination with expert knowledge, we identified 26 sites containing our focal habitat type within the two largest natural habitat complexes in Vojvodina: Fruška Gora and the Deliblato Sands. The two habitat complexes were not treated as categorical predictors because the study focused on continuous environmental gradients rather than explicit regional comparisons.

**Figure 1:**
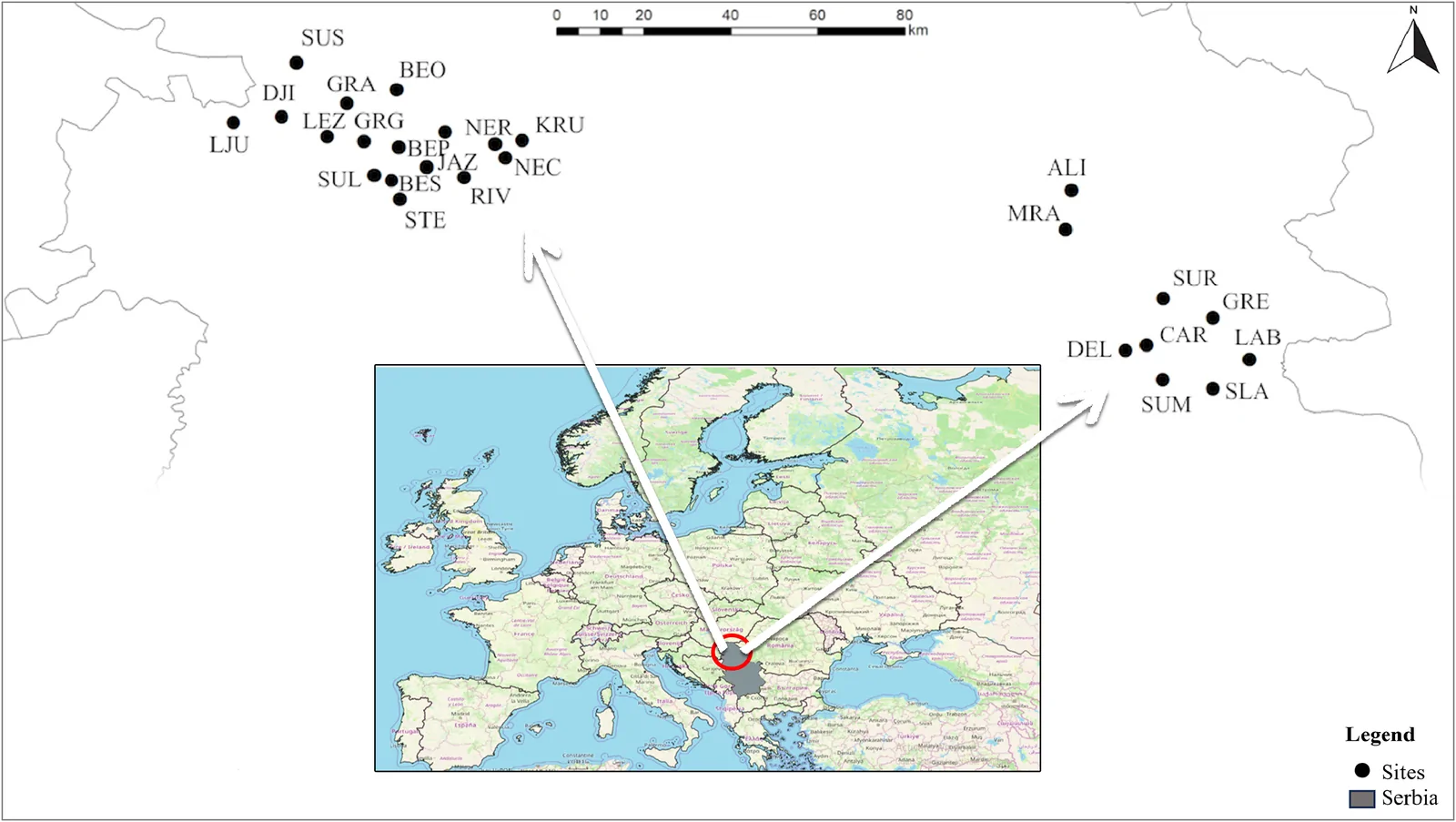
Map of 26 sites in the province of Vojvodina, Serbia, where the sampling of pollinators was conducted. BEO- Beočin, SUS- Susek, LEZ- Ležimir, GRG- Grgurevci, STE- Stejanovci, SUL- Šuljam, LJU- Ljuba, DJI- Ðipša, KRU- Krušedol, NER- Neradin, RIV- Rivica, VRD- Vrdnik, JAZ- Jazak, BES- Bešenovo, BEP- Bešenovački Prnjavor, GRA- Grabovo, NEC- Neradin-česma, ALI- Alibunar, MRA- Mramorak, GRE- Grebenac, SUR- Šušara, SUM- Šumarak, LAB- Labudovo okno, SLA- Slatina, CAR- Čardak, DEL- Deliblato. OpenStreetMap in QGIS was used to create the map of Europe (View → Panels → Browser → OpenStreetMap). Country borders were obtained from: https://developers.google.com/earth-engine/datasets/catalog/FAO_GAUL_2015_level0?hl=es-419 (FAO GAUL: Global Administrative Unit Layers, 2015).

To ensure temporal coverage across the growing season, sampling was conducted throughout 2022 during three periods: spring (April–May), summer (June), and autumn (August–September). Field sampling was approved by the Ministry of Environmental Protection of the Republic of Serbia (license approval number: 353-01-155/2022-04).

Each site was surveyed once per season, resulting in three visits per site. Species richness was calculated as the cumulative (pooled) number of unique species detected across the three seasonal surveys at each site, such that species recorded in multiple surveys were counted only once. Total abundance was summed across the three seasonal surveys to obtain one cumulative annual value per site and taxon. This approach was used because the study focused on overall site-level patterns rather than seasonal dynamics. All statistical analyses were therefore conducted at the site level (*n* = 26), and the final dataset did not contain a repeated-measures structure.

We recorded all individuals of the focal taxa (wild bees, hoverflies, and butterflies) observed within the predefined transect corridor, regardless of whether they were actively feeding on flowers or just flying within the transect area. Individuals merely passing outside the transect boundaries were not recorded. Observations were conducted between 9:00 and 17:00 under thresholded weather conditions (air temperature ≥ 13 °C on sunny days and ≥ 17 °C on cloudy days) (Pollard & Yates, 1993; Westphal et al., 2008; Van Swaay & Warren, 2012). Two transects were established per site. Wild bees and hoverflies were surveyed along a shared transect which was 500 m long and 2 m wide (1 m on each side of the observer), as both taxa require close-range detection and frequent netting. Butterflies were surveyed along a separate transect, 600 m long and 5 m wide, following a standard Pollard-walk approach (Pollard & Yates, 1993), which requires a wider observation belt. Separate transects ensured methodological consistency for each taxon while maintaining comparable habitat conditions.

Both transects were located within the same semi-arid grassland patch and placed in close proximity to ensure comparable habitat conditions without spatial overlap. Each transect was surveyed for 30 min and divided into 5-minute sections (approximately 83 m for bees/hoverflies and 100 m for butterflies). Fieldwork was carried out simultaneously by two teams to maintain temporal consistency across taxa while avoiding interference between survey methods.

Individuals that could be reliably identified in the field were recorded without capture. Specimens requiring confirmation were collected using entomological nets and subsequently identified in the laboratory using standard taxonomic keys. Identification followed established literature: for wild bees, Amiet (1996); for hoverflies, Dušek & Laska (1973), Vujić (1992), Vujić & Šimić (1995-98), Hippa, Nielsen & Steenis (2001), Van Veen (2004), Vujić et al. (2013), Mengual et al. (2015), Speight & Sarthou (2017), Tot (2021), and Vujić et al. (2021); and for butterflies, Popović & Ðurić (2011). Surveys were conducted by trained specialists for each taxonomic group to ensure high identification accuracy.

### Transect characteristics

Total flower cover was visually estimated along the entire transect length as the percentage of the transect area occupied by flowers of all flowering plant species, calculated as the flower-covered area divided by the total transect area and multiplied by 100. Total flower cover was estimated jointly by two trained observers following a standardized protocol, and values were agreed upon in the field to minimize inter-observer variability. Vegetation height was measured as the mean height of the herbaceous layer along each transect (cm). Measurements were taken at three standardized points (beginning, middle, and end of the transect) using a measuring tape, and their average was used as the representative vegetation height for that transect. Green vegetation cover (%) was visually estimated as the proportion of each transect occupied by green vegetation, calculated as the vegetated area divided by the total transect area and expressed as a percentage. All transect-level variables were recorded during each seasonal survey and subsequently averaged across seasons to obtain a single mean value per site.

**Land cover characteristics**: The percentage cover (%) of land cover types, agricultural, forest, and grassland was calculated within a 1 km radius using Corine Land Cover maps (EEA 2020) and QGIS (QGIS 3.28).

**Weather variables**: were recorded during each sampling event at the begnining of the transect walk. Air temperature (^∘^C) and wind speed (m s^−^^1^) were measured in the field using standard portable automatic instruments. Surveys were not conducted during rainfall or persistent precipitation events. Cloud cover was visually estimated as the percentage (%) of sky covered by clouds at the time of sampling. Sunshine duration was estimated as the percentage (%) of the transect walk carried out during sunshine, and rounded to the nearest 10%. To ensure consistency with the site-level biological data, weather variables were averaged across the three seasonal surveys at each site to represent mean sampling conditions.

### Data analysis

#### Multivariate patterns of community composition

To investigate the influence of landscape and transect variables on species composition for each taxon separately, we applied Redundancy Analysis (RDA), after checking the gradient length with Detrended correspondence analysis (DCA) to justify a linear method (Legendre & Legendre, 1998). As RDA was designed to evaluate how landscape- and transect-level habitat variables structure species composition across sites, short-term weather variables were not included in this analysis. Weather conditions primarily influence short-term activity and detectability during sampling, rather than long-term community assembly patterns.

Prior to ordination, environmental predictors were screened for multicollinearity using the VIF function (all VIFs < 5; Zuur, Ieno & Elphick, 2010). Species data were Hellinger-transformed to downweight rare species (Legendre & Gallagher, 2001). The overall significance of the RDA model was evaluated by a permutation test with 999 randomizations (*p* < 0.05), and the significance of individual environmental variables through separate permutation tests (*p* < 0.05).

### Environmental drivers of pollinator abundance and richness

To assess taxon-specific responses we applied generalized additive models (GAMs; Wood, 2006) separately for each pollinator group. For each taxon, two response variables were analyzed, observed species richness and total abundance. Because observed species richness can be constrained by the number of individuals recorded, we assessed the relationship between observed richness and total abundance using Spearman’s rank correlations. We also conducted sensitivity analyses using alternative diversity metrics less directly dependent on abundance. Fisher’s alpha (Fisher, Corbet & Williams, 1943) was used for wild bees and butterflies, while Shannon diversity was used for hoverflies because Fisher’s alpha produced unstable estimates for this taxon. These metrics were modelled using the same GAM framework and predictor groups as the observed-richness models. To reflect ecological scale and avoid overparameterization, environmental predictors were grouped into three categories and fitted in separate models: (1) landscape-scale variables: grassland cover, forest cover, and agricultural land cover, (2): transect-scale habitat variables: total flower cover, vegetation height, and green vegetation cover, and (3): weather variables: cloud cover, sunshine duration, air temperature, and wind speed. Each predictor group was analyzed in a separate GAM to facilitate interpretation and to avoid strong collinearity across spatial scales. Predictor variables were analyzed in their original units without standardization, as scaling does not affect inference in GAMs (Wood, 2017), and retaining original units facilitates ecological interpretation.

Species richness was modeled using a Poisson error distribution with a log link function. Abundance data were modeled using a negative binomial distribution with a log link to account for overdispersion. Fisher’s alpha and Shannon diversity were modelled using Gaussian GAMs. All continuous predictors were fitted as smoothed terms using cubic regression splines with shrinkage (bs = "cs"). This approach allows smoothed terms to be penalized toward zero when predictors provide little explanatory power, thereby reducing overfitting and avoiding post-hoc variable selection. The basis dimension was set to *k* = 4 for all smoothed terms, reflecting the relatively small sample size (*n* = 26) and the expectation of simple, monotonic or weakly nonlinear relationships (Wood, 2010). Models were fitted using restricted maximum likelihood (REML). Model summaries were inspected to assess the significance of smoothed terms using approximate chi-square tests. Model fit was evaluated using adjusted R^2^ and deviance explained.

The adequacy of the basis dimension (k) was assessed using the k-index diagnostic provided by gam.check() (Wood, 2006; Wood, 2017). No evidence of insufficient basis dimension was detected. For abundance models, residual diagnostics were conducted using the DHARMa package to assess uniformity, dispersion, and the presence of outliers (Hartig, 2024).

To further evaluate the influence of sample size and sampling completeness on observed richness, we conducted individual-based rarefaction and extrapolation analyses. Rarefaction curves were calculated separately for each pollinator taxon using abundance-based community matrices and Hill number order *q* = 0, corresponding to species richness. We also estimated sample coverage for each taxon and site to assess sampling completeness. These analyses were used as supplementary diagnostics to evaluate whether differences in observed richness could reflect differences in the number of individuals sampled or incomplete sampling. Based on these results, observed richness was interpreted cautiously and complemented with alternative diversity metrics less directly dependent on abundance.

To assess potential spatial autocorrelation among sampling sites, Moran’s I tests (Moran, 1950) were applied to Pearson residuals of representative generalized additive models (GAMs).

All analyses were conducted in R 4.3.2 (R Core Team, 2023) using the packages: car for VIFs check (Fox & Weisberg, 2018), vegan for DCA and RDA (Oksanen et al., 2024), mgcv for GAMs (Wood, 2006; Wood, 2017), DHARMa for residual diagnostics and Moran’s I spatial tests (Hartig, 2024), iNEXT for rarefaction and sample-coverage analyses (Hsieh, Ma & Chao, 2016).

## Results

### Multivariate patterns of community composition

We identified a total of 340 pollinator species, surveying 7,822 pollinator specimens (Table S1), which included 1,807 wild bee specimens of 199 species, 1,919 hoverfly specimens of 66 species and 4,096 butterfly specimens of 75 species.

Redundancy analysis (RDA) showed that environmental variables significantly explained variation in community composition for all three pollinator groups (Table S2). The constrained variables accounted for 28.0% of the total variance in wild bee assemblages (*F* = 1.23, *p* = 0.016), 35.5% in hoverflies (*F* = 1.74, *p* = 0.002), and 31.8% in butterflies (*F* = 1.47, *p* = 0.008).

### Wild bees

For wild bees, grassland cover (*F* = 1.629, *p* = 0.010) and agricultural cover (*F* = 1.876, *p* = 0.002) significantly influenced species composition, while forest cover, total flower cover, vegetation height, and green vegetation cover were not significant (Fig. 2A). Projection of species scores onto environmental gradients revealed that grassland-associated assemblages were characterized by species such as *Bombus pascuorum, Andrena oralis, Tetralonia malvae, Halictus rubicundus, Andrena labiata, Dasypoda morawitzi, Megachile centuncularis*. In contrast, agricultural sites were associated with generalist and disturbance-tolerant taxa, including *Halictus (Halictus patellatus)*, *Lasioglossum (Lasioglossum interruptum, Lasioglossum discum)*, *Osmia (Osmia aurulenta)*, *Bombus sylvarum*, and *Bombus terrestris.*

### Hoverflies

For hoverflies, community composition was primarily structured along an agricultural–forest gradient, with agricultural (*p* = 0.002) and forest cover (*p* = 0.044) as significant predictors. Grassland cover showed a marginal effect (*p* = 0.067; Fig. 2B).

Projection of species scores onto the agriculture-forest gradient showed that *Eristalis arbustorum* and *Eristalis tenax*, *Eristalinus aeneus*, *Helophilus trivittatus*, were most strongly associated with agricultural sites, whereas *Merodon aberrans, Sphaerophoria scripta,* and *Merodon moenium, Eupeodes corollae, Pipizella viduata* were most strongly associated with forest-dominated sites.

**Figure 2:**
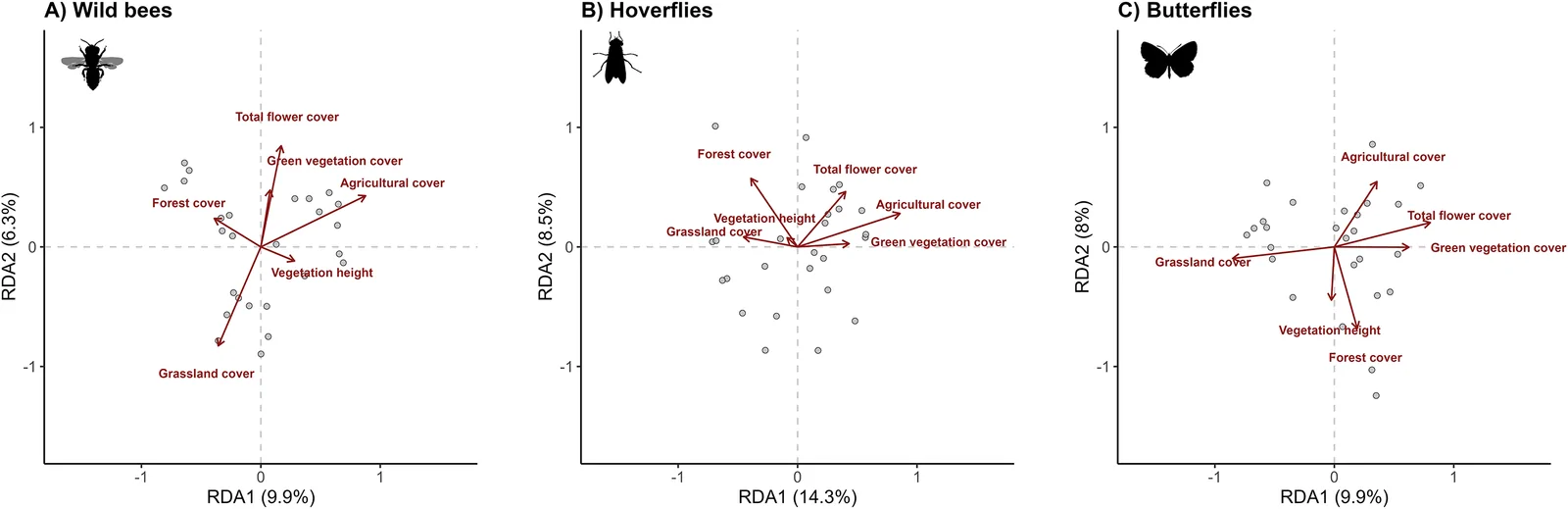
Redundancy analysis (RDA) of pollinator community composition in relation to environmental variables. Panels show (A) wild bees, (B) hoverflies, and (C) butterflies. Points represent sampling sites and arrows denote environmental predictors. Axis percentages indicate explained variance. All models were significant (*p* < 0.05). Grassland and agricultural cover significantly structured wild bee communities; agricultural and forest cover structured hoverfly communities; and grassland cover structured butterfly communities. Species names are omitted for clarity. Image Source Credits:- *Megachile rotundata*: Melissa Broussard, https://www.phylopic.org/images/5e16fc5c-8bdb-44b3-91cc-6805ee5eba1d/megachile-rotundata, CC BY 3.0 Unported- *Eristalis tenax*: Gareth Monger, https://www.phylopic.org/images/fdd9c1c9-7391-4e2b-af4e-a0d289fa9466/eristalis-tenax, CC BY 3.0 Unported- *Plebejus argus*: Vítězslav Maňák, https://www.phylopic.org/images/04af22bd-6772-445e-a66f-1009173eea2d/plebejus-argus, CC BY 4.0.

### Butterflies

For butterflies, only grassland cover (*p* = 0.005) significantly explained variation in species composition, whereas all other environmental variables were non-significant (Fig. 2C). Species scores projected onto the grassland cover gradient identified *Issoria lathonia*, *Coenonympha pamphilus*, *Hipparchia statilinus*, *Maniola jurtina*, and *Argynnis pandora* as species most strongly associated with grassland-rich sites.

### Environmental drivers of pollinator abundance and richness

Observed species richness was significantly correlated with total abundance in all three pollinator taxa: wild bees (Spearman’s *ρ* = 0.58, *p* = 0.0018), hoverflies (*ρ* = 0.60, *p* = 0.0013), and butterflies (*ρ* = 0.48, *p* = 0.012). In contrast, the alternative diversity metrics were not significantly correlated with abundance: Fisher’s alpha for wild bees (*ρ* = 0.15, *p* = 0.478), Shannon diversity for hoverflies (*ρ* = 0.07, *p* = 0.730), and Fisher’s alpha for butterflies (*ρ* = −0.12, *p* = 0.556). Therefore, observed-richness results were evaluated together with the alternative diversity metrics.

### Wild bees

Wild-bee abundance was not significantly affected by any examined landscape, transect, or weather predictor. All smoothed terms across models had estimated degrees of freedom close to zero and non-significant test statistics (*p* > 0.11). Deviance explained was negligible for landscape and transect models (<1%), and modest for the weather model (23%), driven by weak, non-significant trends.

Observed wild-bee richness declined with grassland cover in the landscape model (edf =2.79, *χ*^2^ = 27.60, *p* < 0.001; Fig. 3A), which explained 40.8% of deviance. No transect-scale predictor was significantly associated with observed wild-bee richness, and the model explained very little variation. In the weather model, temperature (edf =1.47, *χ*^2^ = 11.94, *p* < 0.001; Fig. 3B) and wind speed (edf =2.34, *χ*^2^ = 13.51, *p* < 0.001; Fig. 3C; Table S3A) were significantly associated with observed wild-bee richness, with the weather model explaining 40.9% of deviance.

**Figure 3:**
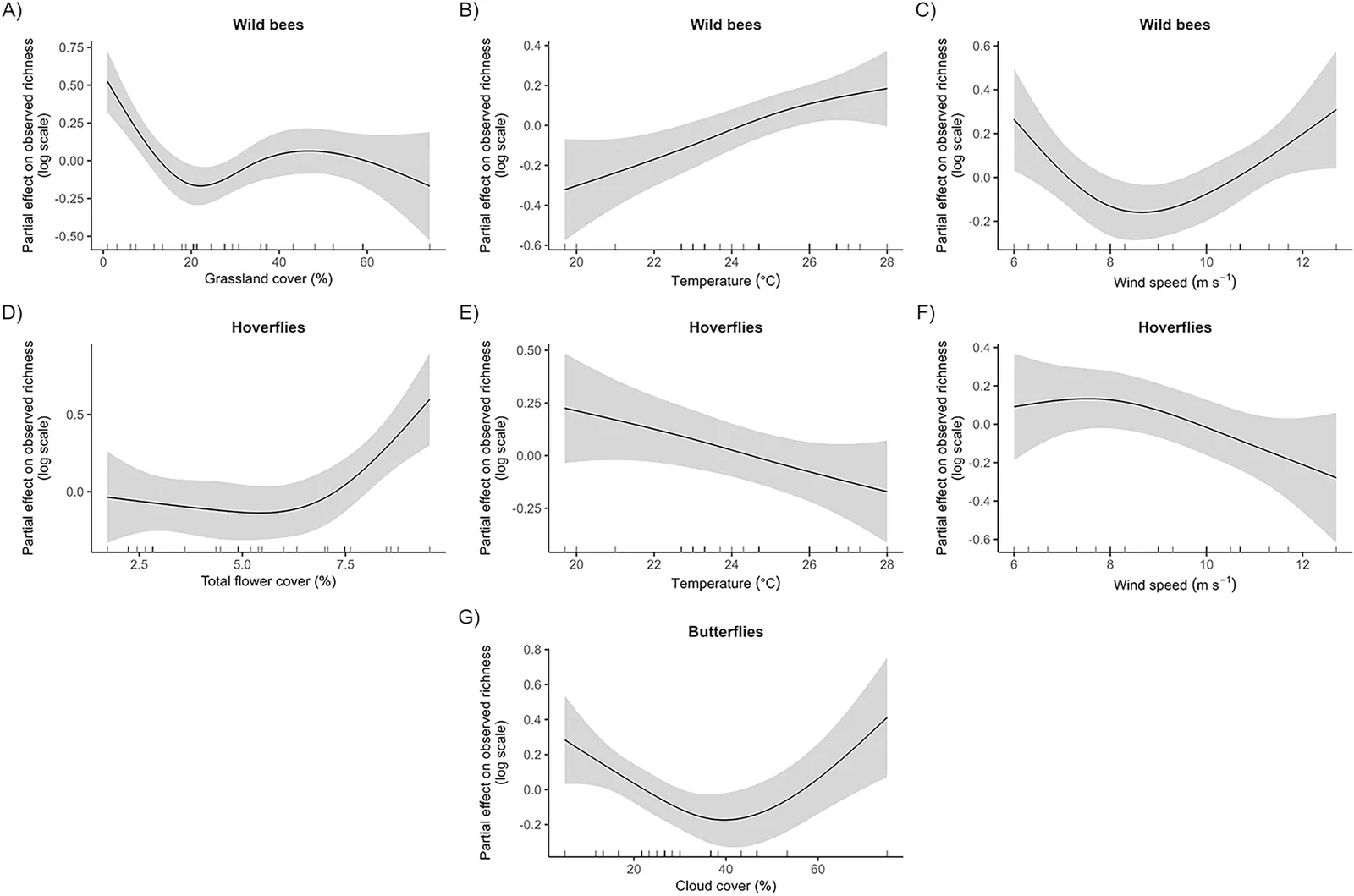
Generalized additive model (GAM) smooths showing significant effects of environmental predictors on observed pollinator species richness for wild bees (A–C), hoverflies (D–F), and butterflies (G). Solid lines represent estimated smooth functions, shaded areas indicate 95% confidence intervals, and rug plots show the distribution of observations along predictor gradients. Predictor variables are shown in their original measurement units: grassland cover, total flower cover, and cloud cover as percentages (%); temperature in °C; and wind speed in ms^−1^. Observed richness represents cumulative annual richness detected across the three seasonal sampling sessions.

In the Fisher’s alpha sensitivity models, the effect of grassland cover was not retained (*p* = 0.986). Wild-bee Fisher’s alpha was significantly associated with vegetation height (edf =2.09, *F* = 3.58, *p* = 0.0097; Fig. S1A), and the transect-scale model explained 35.9% of deviance. The significant temperature and wind effects observed for species richness were also not retained in the Fisher’s alpha models.

### Hoverflies

Hoverfly abundance showed clear responses to local habitat structure, but not to weather. At the landscape scale, abundance decreased with grassland cover (edf =1.04, *χ*^2^ = 5.99, *p* = 0.008, Fig. 4A), explaining 20.6% of the deviance. At the transect scale, total flower cover was significant positive predictor of abundance (edf =1.13, *χ*^2^ = 7.92, *p* = 0.003, Fig. 4B), explaining 25.3% of the deviance. In contrast, none of the weather variables significantly affected hoverfly abundance (deviance explained <1%; Table S3B).

**Figure 4:**
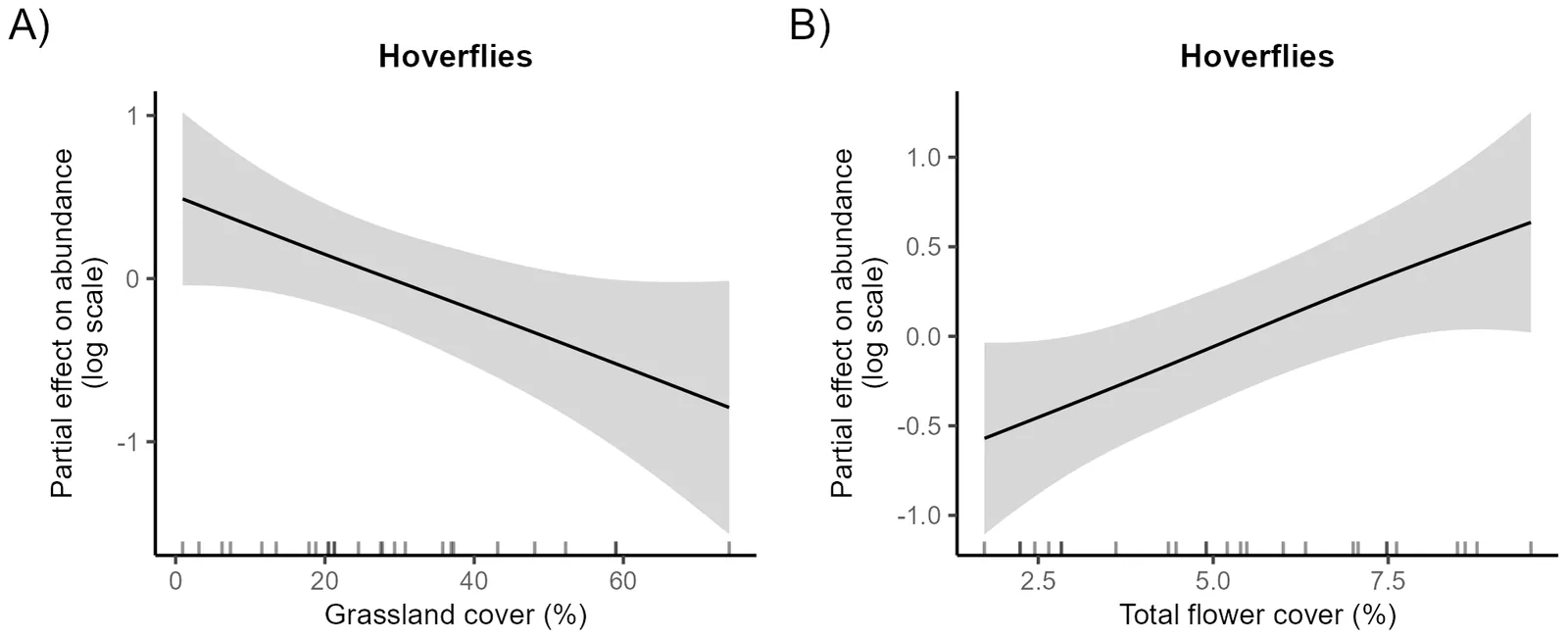
Generalized additive model (GAM) smooths showing the effects of landscape and transect-scale predictors on hoverfly abundance. (A) shows the effect of grassland cover (%), while (B) shows the effect of total flower cover (%). Shaded areas represent 95% confidence intervals and rug plots indicate the distribution of observations along predictor gradients. Abundance values represent cumulative annual abundance across the three seasonal sampling sessions.

No landscape-scale predictor was significantly associated with observed hoverfly richness. At the transect scale, observed hoverfly richness was significantly associated with total flower cover (edf =2.14, *χ*^2^ = 16.04, *p* < 0.001; Fig. 3D), and the transect model explained 35.3% of deviance.

Temperature (edf =1.00, *χ*^2^ = 4.38, *p* = 0.021; Fig. 3E) and wind speed (edf =1.45, *χ*^2^ = 5.48, *p* = 0.022; Fig. 3F) were significantly associated with observed hoverfly richness.

In contrast, Shannon diversity showed no significant relationship with total flower cover (*p* = 0.928), but did show weak marginal relationships with ground cover (edf =0.81, *F* = 1.00, *p* = 0.055) and temperature (edf =0.88, *F* = 0.83, *p* = 0.088), with no significant effects at *p* < 0.05.

### Butterflies

No significant effects of landscape, transect, or weather variables on butterfly abundance were detected. Forest cover showed a weak positive trend (*p* = 0.095), whereas all other predictors were non-significant.

No landscape-scale or transect-scale predictor was significantly associated with observed butterfly richness. Observed butterfly richness was significantly associated with cloud cover (edf =2.25, *χ*^2^ = 14.44, *p* < 0.001; Fig. 3G), and the weather model explained 33.5% of deviance (Table S3C). This result was supported by the Fisher’s alpha sensitivity model, in which cloud cover was also significant (*edf* = 0.97, *F* = 1.18, *p* = 0.049; Fig. S1B). No landscape or transect effects were detected for butterfly Fisher’s alpha.

### Model diagnostics and sampling completeness

Simulation-based residual diagnostics indicated no significant deviations from uniformity, dispersion, or outlier expectations for all abundance models (all *p* > 0.05), suggesting adequate model fit. Across all fitted models, Moran’s I tests indicated no significant spatial autocorrelation in the residuals (all *p* > 0.05). This pattern was consistent across taxa and response variables, suggesting that spatial structure was adequately accounted for at the scale of analysis.

Rarefaction and sample-coverage analyses indicated that observed richness was influenced by the number of individuals sampled and by differences in sampling completeness among taxa and sites (Figs. S2 and S3). Coverage was highest for butterflies, generally high but more variable for hoverflies, and lowest and most variable for wild bees (Fig. S3). These results support the cautious interpretation of observed richness and the use of Fisher’s alpha for wild bees and butterflies and Shannon diversity for hoverflies as complementary sensitivity analyses.

## Discussion

### Multivariate patterns of community composition

#### Wild bees

Wild bee composition varied primarily along the agriculture–grassland gradient, indicating strong landscape structuring of community change. Grassland-dominated sites were characterized by species associated with open, semi-natural habitats with heterogeneous vegetation structure and diverse floral resources. Among these taxa, several species show clear habitat preferences, including specialization on particular host plants (*e.g.*, *Tetralonia malvae* feeding on Malvaceae; Dorchin & Michez, 2024) and sandy substrates in dry grasslands (*e.g.*, *Dasypoda* spp.; Radchenko, 2016). In contrast, agricultural sites were dominated by more generalist and disturbance-tolerant taxa, consistent with simplified habitat conditions and reduced resource diversity. Many of these species possess ecological traits that facilitate persistence in managed landscapes, including broad dietary breadth, flexible nesting strategies, and high dispersal capacity (Kasina et al., 2009; Martins, 2008; Nelson et al., 2023). Social bumblebees and small halictids are known for their adaptability to agricultural mosaics and mass-flowering crops. Although mechanisms were not directly tested, change along the agriculture–grassland gradient may reflect differences in nesting opportunities, floral continuity, and land-use intensity.

### Hoverflies

Hoverfly assemblages were structured along the agricultural–forest gradient, likely reflecting larval habitat requirements and the ability of mobile species to track resource patches across agricultural landscape (Doyle et al., 2020; Branquart & Hemptinne, 2000; Lucas et al., 2018). Agricultural sites were primarily associated with *Eristalis arbustorum*, *Eristalis tenax*, *Eristalinus aeneus*, and *Helophilus trivittatus*, species that exploit nutrient-rich, disturbed habitats and frequently breed in organically enriched substrates such as manure, slurry, ditches, and eutrophic water bodies typical of agricultural landscapes (Pérez-Bañón et al., 2013; Devriese et al., 2025). At the forest end of the gradient, *Merodon aberrans*, *M. moenium*, and *Pipizella viduata* were more prominent. Species of *Merodon* are often associated with semi-natural habitats where their bulb-feeding larvae depend on specific host plants, while *Pipizella* species frequently occur in structurally complex habitats that support aphid populations and provide sheltered microclimates (Verlinden, 1999). Generalist aphidophagous species such as *Sphaerophoria scripta* and *Eupeodes corollae*, which are well documented from agricultural landscapes, also projected toward the forest end of the gradient. Although both species are commonly associated with crop systems due to their role as aphid predators (Hodgkiss, Brown & Fountain, 2018; Pekas et al., 2020), they are frequently recorded along deciduous forest edges and at field–woodland boundaries within agricultural mosaics (Bennewicz, 2011; Gaytán, Ricarte & González-Bornay, 2020; Burgio et al., 2025). Their position likely reflects responses to increased habitat heterogeneity or reduced agricultural intensity rather than strict forest specialization.

### Butterflies

Species associated with grassland-rich sites included *Issoria lathonia*, *Coenonympha pamphilus*, *Hipparchia statilinus*, *Maniola jurtina*, and *Argynnis pandora*, all characteristic of sun-exposed grasslands. These species rely on specific larval host plants (*e.g.*, *Viola* spp. for *I. lathonia* and *A. pandora*, dry steppe grasses such as *Festuca ovina*, *Deschampsia cespitosa*, and *Corynephorus canescens* for *H. statilinus*, or common grasses for *M. jurtina* and *C. pamphilus*), as well as suitable vegetation structure (Van Swaay, 2002; Middleton-Welling et al., 2020; Van Swaay et al., 2020).

### Environmental drivers of pollinator abundance and richness

#### Wild bees

Wild-bee abundance showed no consistent response to landscape, local habitat, or weather variables, suggesting that additional factors not measured here, such as nesting substrate availability, grassland management, or seasonal resource continuity, may influence local population density.

Observed wild bee richness declined with increasing grassland cover, perhaps indicating that landscape heterogeneity rather than grassland dominance per se promotes diversity. Heterogeneous mosaics can provide complementary nesting and floral resources (Neokosmidis et al., 2016; Eckerter et al., 2022; Parreno et al., 2024), whereas landscapes dominated by single habitat type may offer fewer resource types or reduced structural variation. In intensively cultivated regions such as our study area, grasslands can differ substantially in management intensity, plant diversity, and structural complexity. Increasing land-use intensity has been shown to constrain wild bee richness even within grassland systems (Ekroos et al., 2019). Because management intensity and habitat quality were not directly measured, grassland extent alone cannot be interpreted as a proxy for habitat suitability (Albrecht et al., 2023; Schlager et al., 2024). Thus, the observed relationship should be considered context dependent and potentially specific to agricultural landscapes where grasslands are embedded within intensively managed matrices. Taking into consideration that grassland effect was not retained in the Fisher’s alpha sensitivity model, this relationship should be interpreted as a pattern in detected richness, potentially influenced by abundance, activity, or sampling completeness, rather than as clear evidence of abundance-independent diversity change.

Weather also influenced observed wild bee richness. Observed richness increased with temperature, consistent with previous findings that warmer conditions enhance flight activity and detectability (Burdine & McCluney, 2019; Welti et al., 2021; Geppert et al., 2022). The nonlinear response to wind likely reflects activity-mediated processes rather than a simple ecological effect. Wind affects flight stability and energetic cost and may also correlate with site exposure and vegetation structure (Burnett, Badger & Combes, 2022). Moreover, bee species differ in their tolerance to wind due to variation in body size, wing loading, and flight morphology (Combes & Dudley, 2009), which may lead to differential activity patterns under varying wind conditions.

Since weather effects were also not retained in the Fisher’s alpha models, it suggests that these results reflect variation in activity or detectability rather than abundance-independent diversity. In contrast, Fisher’s alpha being significantly related to vegetation height, indicates that local vegetation structure may be more relevant for abundance-independent wild-bee diversity than the landscape and weather predictors that explained observed richness. Vegetation height may reflect management intensity, microclimatic conditions, or structural complexity relevant to foraging and nesting (Main et al., 2019).

### Hoverflies

Observed hoverfly richness and abundance were primarily driven by local floral resources rather than landscape context at the scale we measured (1 km). Increasing floral cover strongly enhanced observed richness, consistent with adult dependance on nectar and pollen (Kleijn & Van Langevelde, 2006; Ebeling et al., 2008; Lucas et al., 2017). The threshold-like pattern suggests potential resource limitation at lower flower densities. However, this flower-cover effect was not retained in the Shannon diversity sensitivity model. Because observed hoverfly richness was correlated with abundance whereas Shannon diversity was not, the richness response to flower cover likely reflects increased abundance, local attraction, or detectability in flower-rich transects rather than a clear increase in abundance-independent diversity.

Weather negatively influenced observed richness, with declines under high temperature and wind, likely reflecting activity-mediated detectability. The negative association with temperature may reflect activity-related constraints during sampling, such as reduced flight activity or increased desiccation risk under warmer conditions. Similarly, wind is known to impair flight efficiency and foraging behavior in flying insects, including hoverflies (Balfour & Ratnieks, 2025), which may reduce the number of species active and detectable under windy conditions.

As with flower cover, these weather effects were not retained in the Shannon diversity models, and Shannon diversity showed only marginal relationships with ground cover and temperature, suggesting that observed hoverfly richness was more strongly linked to abundance or activity patterns than to abundance-independent diversity.

Hoverfly abundance responded more strongly to habitat structure, increasing with flower cover and decreasing with grassland extent. Since grassland quality and management intensity were not directly assessed, this relationship should be interpreted cautiously. The negative association with grassland extent may reflect differences in landscape heterogeneity or resource distribution across sites. In agricultural landscapes, mosaics of crop fields, field margins, and semi-natural elements, can provide diverse adult foraging opportunities and larval substrates (Schweiger et al., 2007; Burgio & Sommaggio, 2007; Hendrickx et al., 2007; Meyer, Jauker & Steffan-Dewenter, 2009).

### Butterflies

Observed butterfly richness was primarily influenced by cloud cover. Despite thresholded survey conditions (Pollard & Yates, 1993), residual variation in cloud cover affected activity and detectability. Butterflies are highly sensitive to solar radiation, and even moderate differences in cloud cover can alter activity levels and detectability (Wikström, Milberg & Bergman, 2009; Cormont et al., 2011). The U-shaped relationship likely reflects context-dependent thermoregulatory constraints, because cloud cover interacts with ambient temperature (Riva et al., 2020). Unlike the richness patterns observed for wild bees and hoverflies, the butterfly response to cloud cover was retained in the Fisher’s alpha sensitivity model. Since butterfly Fisher’s alpha was not correlated with abundance, this relationship suggests a more robust diversity-related response.

Compared to bees and hoverflies, weaker structural habitat effects on butterflies may reflect higher mobility (Hausharter et al., 2023; Franzén et al., 2024) and responses operating at broader spatial scales (Brückmann, Krauss & Steffan-Dewenter, 2010; Ferrer-Paris et al., 2013).

Given the moderate sample size, the analyses were intentionally conservative, and weaker effects may have remained undetected. Consequently, the significant relationships reported here are likely to represent the strongest environmental drivers operating across the study system. In addition, because the analyses were conducted across the full monitoring period, seasonal differences in pollinator activity may have contributed to variation in weather responses. Future studies with larger sample sizes within individual seasons could explicitly test whether the strength and direction of weather effects differ among seasons.

## Conclusions

Our results demonstrate that pollinator responses are taxon-specific and differ between broader landscape context and local environmental conditions. Across all three taxa, species composition was driven primarily by landscape-scale variables, indicating that landscape context plays an important role in determining which species occur, whereas local habitat features and short-term weather conditions mainly influence observed richness, abundance, and activity-related patterns. Weather significantly affected observed richness despite thresholded sampling, demonstrating that biologically meaningful variation in pollinator activity can persist even within accepted monitoring conditions.

Sensitivity analyses further highlighted the importance of distinguishing observed species richness from abundance-independent diversity. Observed richness was positively correlated with total abundance in all three taxa, indicating that observed richness patterns partly reflected variation in the number of individuals detected. Several relationships identified using observed richness weakened when alternative diversity metrics were applied, particularly for wild bees and hoverflies, suggesting that some richness responses reflected abundance, activity, or detectability rather than diversity structure alone. In contrast, the butterfly response to cloud cover remained consistent across both observed richness and Fisher’s alpha.

Although this study was conducted in a semi-arid agricultural landscape in Serbia and was based on a single field season, the findings are likely relevant to other intensively managed systems where pollinator communities respond to landscape heterogeneity, local resource availability, and short-term climatic conditions. The results should therefore be interpreted as within-season spatial relationships rather than long-term temporal trends.

Overall, our study highlights the importance of integrating multi-scale habitat variables, weather covariates, and complementary diversity metrics in pollinator monitoring and conservation research, and may help inform biodiversity measures within agricultural policy frameworks such as the EU Common Agricultural Policy.

##  Supplemental Information

10.7717/peerj.21629/supp-1 Supplemental Information 1 Sensitivity analyses using alternative diversity metrics Panel A shows the effect of vegetation height (mm) on wild bee Fisher’s alpha diversity, while panel B shows the effect of cloud cover (%) on butterfly Fisher’s alpha diversity. Shaded areas represent 95% confidence intervals and rug plots indicate the distribution of observations along predictor gradients.

10.7717/peerj.21629/supp-2 Supplemental Information 2 Sample-size-based rarefaction and extrapolation curves for pollinator communities across study sites Panels show species richness estimates for (A) wild bees, (B) hoverflies, and (C) butterflies. Solid lines represent rarefaction (interpolation) based on observed samples, whereas dashed lines represent extrapolated richness estimates beyond the observed sample size. Shaded areas indicate 95% confidence intervals. Symbols denote observed species richness at the reference sample size for each site. Different colours represent individual study sites, illustrating variation in species accumulation patterns and sampling completeness among sites.

10.7717/peerj.21629/supp-3 Supplemental Information 3 Estimated sample coverage for pollinator taxa The sample-coverage plot shows estimated sampling completeness across sites.

10.7717/peerj.21629/supp-4 Supplemental Information 4 Recorded species at surveyed sites

10.7717/peerj.21629/supp-5 Supplemental Information 5 Results of redundancy analysis (RDA) for wild bees, hoverflies, and butterflies. Sequential term tests (999 permutations) are shown for landscape and local habitat variables

10.7717/peerj.21629/supp-6 Supplemental Information 6 Detailed summary of generalized additive models (GAMs) examining environmental drivers of species richness and abundance of wild bees, hoverflies, and butterflies in Vojvodina, Serbia (2020) For each model, reported are model specifications, overall model fit statistics, and significance of smooth terms (edf, *χ*^2^, *p*-values).

## Additional Information and Declarations

### Field Study Permissions

The following information was supplied relating to field study approvals (*i.e.*, approving body and any reference numbers): Field sampling was approved by the Ministry of Environmental Protection of the Republic of Serbia (license approval number: 353-01-155/2022-04).

### Data Availability

The following information was supplied regarding data availability: The data is available at Zenodo: Skendžić, T., & Vujanovic, D. (2026). Multi-scale environmental drivers of pollinator community composition, richness, and abundance across multiple taxa. https://doi.org/10.5281/zenodo.17453701.
