# Supplementary material for: Multi-scale environmental drivers of pollinator community composition, richness, and abundance across multiple taxa
Source: PeerJ. 2026 Sep 30;14:e21629. doi: 10.7717/peerj.21629 (PMC13633686; doi:10.7717/peerj.21629)
Supplement: Supplementary file 1 [file peerj-14-21629-s005.docx]

| A) **Overall model** | | | | |
| --- | --- | --- | --- | --- |
| **Taxon** | **F** | **p** | **% explained variance** | |
| Wild bees | 1.23 | 0.016 | 28% | |
| Hoverflies | 1.74 | 0.002 | 35.5% | |
| Butterflies | 1.47 | 0.008 | 31.8% | |
| B) **Sequential term tests** | | | | |
| **Taxon** | **Variables** | **Variance** | **F** | **p** |
| **Wild bees** | Grassland cover | 0.049 | 1.62 | **0.012** |
|  | Forest cover | 0.027 | 0.90 | 0.681 |
|  | Agricultural cover | 0.056 | 1.87 | **0.002** |
|  | Total flower cover | 0.033 | 1.11 | 0.241 |
|  | Vegetation height | 0.031 | 1.02 | 0.383 |
|  | Green vegetation cover | 0.024 | 0.81 | 0.847 |
| **Hoverflies** | Grassland cover | 0.023 | 1.72 | 0.068 |
|  | Forest cover | 0.025 | 1.83 | **0.038** |
|  | Agricultural cover | 0.041 | 3.05 | **0.003** |
|  | Total flower cover | 0.019 | 1.45 | 0.142 |
|  | Vegetation height | 0.012 | 0.89 | 0.544 |
|  | Green vegetation cover | 0.020 | 1.46 | 0.140 |
| **Butterflies** | Grassland cover | 0.039 | 2.35 | **0.005** |
|  | Forest cover | 0.025 | 1.45 | 0.106 |
|  | Agricultural cover | 0.019 | 1.13 | 0.297 |
|  | Total flower cover | 0.022 | 1.30 | 0.177 |
|  | Vegetation height | 0.021 | 1.25 | 0.185 |
|  | Green vegetation cover | 0.022 | 1.33 | 0.173 |

**Table S2.** Results of redundancy analysis (RDA) for wild bees, hoverflies, and butterflies. Sequential term tests (999 permutations) are shown for landscape and local habitat variables.
